# Validity and reliability of GLIM malnutrition criteria in cardiac surgery

**DOI:** 10.15649/cuidarte.5046

**Published:** 2026-07-07

**Authors:** Mateo Londoño-Pereira, Maite Catalina Agudelo-Cifuentes, Nora Múnera, Sara Paredes, Maritza Londoño, Mónica Yepes

**Affiliations:** 1 Centro Latinoamericano de Nutrición CELAN. Bogotá, Colombia. Departamento de Nutrición Clínica, Clínica Las Américas AUNA. Medellín, Colombia. E-mail: mateolondonop@gmail.com Clínica Las Américas AUNA Medellín Colombia mateolondonop@gmail.com; 2 Facultad de Enfermería, Universidad CES. Medellín, Colombia. E-mail: magudeloc@ces.edu.co Universidad CES Medellín Colombia magudeloc@ces.edu.co; 3 Departamento de Nutrición Clínica, Clínica Las Américas AUNA. Medellín, Colombia. E-mail: noramunera@gmail.com Clínica Las Américas AUNA Medellín Colombia noramunera@gmail.com; 4 Departamento de Nutrición Clínica, Clínica Las Américas AUNA. Medellín, Colombia. E-mail: sara_2583@hotmail.com Clínica Las Américas AUNA Medellín Colombia sara_2583@hotmail.com; 5 Departamento de Nutrición Clínica, Clínica Las Américas AUNA. Medellín, Colombia. E-mail: mari_lode@gmail.com Clínica Las Américas AUNA Medellín Colombia mari_lode@gmail.com; 6 Departamento de Nutrición Clínica, Clínica Las Américas AUNA. Medellín, Colombia. E-mail: monica.yepes@gmail.com Clínica Las Américas AUNA Medellín Colombia monica.yepes@gmail.com

**Keywords:** Protein-Energy Malnutrition, Cardiac Surgical Procedures, Validation Study, Enhanced Recovery After Surgery, Reproducibility of Results, Desnutrición Proteico-Calórica, Procedimientos Quirúrgicos Cardíacos, Estudio de Validación, Recuperación Mejorada Después de la Cirugía, Reproducibilidad de los Resultados, Desnutrição Proteico-Calórica, Procedimentos Cirúrgicos Cardíacos, Estudo de Validação, Recuperação Pós-Cirúrgica Melhorada, Reprodutibilidade dos Testes

## Abstract

**Introduction:**

Malnutrition is common in cardiac surgery patients and it´s associated with adverse clinical outcomes. However, there are no validated criteria for the nutritional diagnosis in this population.

**Objective:**

To determine the criterion validity and inter-rater reliability of the GLIM criteria for the diagnosis of malnutrition and the prediction of outcomes in cardiac surgery.

**Materials and Methods:**

Validation study in adults scheduled for cardiac surgery. Concurrent validity was established with the Subjective Global Assessment and the predictive validity with 30-day hospital readmission. Reliability was assessed with two professionals. The analyses and cut-off points of the GLIM validation guide were followed. Statistical processing was performed in R and Jamovi.

**Results:**

Inter-rater reliability of GLIM was demonstrated, with almost perfect agreement (Kappa 0.94, 95% CI 0.88-0.99, p<0.001), as well as its predictive validity for hospital readmission (RRa 2.59, 95% CI 1.09 - 7.02, p 0.04). Thresholds for concurrent validity were not met (sensitivity 88.0%, CI 95% 68.8-97.5%; specificity 76.9%, CI 95% 56.4-91.0%).

**Discussion:**

The lack of concurrent validity may be explained by differences in muscle mass estimation between diagnostic methods. The results are consistent with previous literature supporting the reliability and predictive ability of GLIM.

**Conclusion:**

GLIM criteria are reliable and predict relevant outcomes in cardiac surgery, supporting their usefulness for nutritional diagnosis and their potential application in the early identification of patients eligible for preoperative optimization interventions.

## Resumen

**Introducción:**

La desnutrición es frecuente en pacientes de cirugía cardiaca y se asocia con desenlaces clínicos adversos. Sin embargo, no existen criterios validados para el diagnóstico nutricional de esta población.

**Objetivo:**

Determinar la validez de criterio y la confiabilidad interevaluador de los criterios GLIM para el diagnóstico de desnutrición y la predicción de desenlaces en cirugía cardiaca.

**Materiales y métodos:**

Estudio de validación en adultos programados para cirugía cardiaca. La validez concurrente se estableció con la Valoración Global Subjetiva y la validez predictiva con el reingreso hospitalario a 30 días. La confiabilidad se evaluó con dos profesionales. Se siguieron los análisis y puntos de corte de la guía de validación de GLIM. El procesamiento estadístico se efectuó en R y Jamovi.

**Resultados:**

Se demostró la confiabilidad interevaluador de GLIM, con concordancia casi perfecta (Kappa 0,94, IC 95% 0,88-0,99, p<0,001), y su validez predictiva para reingreso hospitalario (RRa 2,59, IC 95% 1,09 – 7,02, p 0,04). No se alcanzaron los umbrales de validación concurrente (sensibilidad 88,0%, IC 95% 68,8-97,5%; especificidad 76,9%, IC 95% 56,4-91,0%).

**Discusión:**

La falta de validación concurrente podría explicarse por diferencias en la estimación de la masa muscular entre métodos diagnósticos. Los resultados coinciden con literatura previa que respalda la confiabilidad y capacidad predictiva de GLIM.

**Conclusiones:**

Los criterios GLIM son confiables y predicen desenlaces relevantes en cirugía cardiaca, lo que respalda su utilidad para el diagnóstico nutricional y su potencial aplicación en la identificación temprana de pacientes candidatos a intervenciones de optimización preoperatoria.

## Resumo

**Introdução:**

A desnutrição é frequente em pacientes submetidos à cirurgia cardíaca e está associada a desfechos clínicos adversos. No entanto, não existem critérios validados para o diagnóstico nutricional nessa população.

**Objetivo:**

Determinar a validade de critério e a confiabilidade entre avaliadores dos critérios GLIM para o diagnóstico de desnutrição e a predição de desfechos em cirurgia cardíaca.

**Materiais e métodos:**

Estudo de validação realizado em adultos candidatos à cirurgia cardíaca. A validade concorrente foi estabelecida com a Avaliação Global Subjetiva e a validade preditiva com a readmissão hospitalar em 30 dias. A confiabilidade foi avaliada por dois profissionais. As análises e pontos de corte seguiram o guia de validação dos critérios GLIM. O processamento estatístico foi realizado nos programas R e Jamovi.

**Resultados:**

Demonstrou-se a confiabilidade entre avaliadores dos critérios GLIM, com concordância quase perfeita (Kappa 0,94, IC 95% 0,88–0,99, p<0,001), e sua validade preditiva para readmissão hospitalar (RRa 2,59, IC 95% 1,09–7,02, p 0,04). Não foram alcançados os limiares de validade concorrente (sensibilidade 88,0%, IC 95% 68,8–97,5%; especificidade 76,9%, IC 95% 56,4–91,0%).

**Discussão:**

A ausência de validade concorrente pode ser explicada por diferenças na estimativa da massa muscular entre métodos diagnósticos. Os resultados são consistentes com a literatura prévia que respalda a confiabilidade e a capacidade preditiva dos critérios GLIM.

**Conclusões:**

Os critérios GLIM são confiáveis e predizem desfechos relevantes em cirurgia cardíaca, apoiando sua utilidade para o diagnóstico nutricional e seu potencial na identificação precoce de pacientes candidatos a intervenções de otimização pré-operatória.

## Introduction

**Disease-related malnutrition**

Malnutrition is defined as an imbalance between the formation and breakdown of body tissues and nutrient reserves, leading to loss of muscle and organ mass, decreased physical and mental function, and unfavorable clinical outcomes^1^. In particular, disease-related malnutrition (DRM) results from reduced nutrient intake or absorption caused by acute or chronic diseases, with or without an associated systemic inflammatory response^2^. Its average global prevalence is estimated at 30.9%^3^, and in Latin America, prevalences ranging from 40% to 60% have been reported in hospital settings^4^. Given its magnitude and relevance, the World Health Organization (WHO) recently approved codes 5B72.0 and 5B72.1 in the International Classification of Diseases, 11th Revision (ICD-11), officially recognizing DRM as a pathological entity^5^.

**Disease-related malnutrition in cardiac surgery**

Patients undergoing cardiac surgery have a greater predisposition to DRM, as a consequence of the pathophysiological response to the underlying disease and surgical trauma, characterized by neurohormonal, metabolic, immunological, and inflammatory alterations that lead to the activation of proteolytic pathways and muscle wasting, anorexia, early satiety, malabsorption, and reduced functional capacity^6^,^7^. Malnutrition in this patient group is associated with a higher risk of cardiac and gastrointestinal complications, prolonged hospital stays, greater need for antibiotic therapy and vasopressor support, increased healthcare costs, and a lower overall survival rate^8^–^11^. Although recognized as a high-risk nutritional and clinical group, these patients have the highest rates of iatrogenic malnutrition^12^,^13^. This is possibly due to the lack of validated criteria for their nutritional diagnosis^14^, following the precept that what is not detected cannot be treated.

**Global Leadership Initiative on Malnutrition**

The Global Leadership Initiative on Malnutrition (GLIM) was launched in 2016 with the participation of multiple scientific societies of clinical nutrition from Europe, Asia, North America and South America. In 2019 the official document of the initiative was published, presenting the diagnostic criteria for DRM^15^, which are intended to be universally accepted and allow for the standardization of malnutrition diagnosis. The GLIM criteria have been translated into Spanish and have face and content validity as they come from expert judgment; however, the authors point out that their criterion validity and reproducibility in different populations and clinical groups need to be evaluated before their incorporation into medical practice^16^.

**Gaps in literature**

A systematic review identified more than 61 criterion validation studies of the GLIM criteria; however, none included patients undergoing cardiac surgery^17^. To the best of our knowledge, only Liu et al.^11^ have evaluated the GLIM criteria in this population, in an Asian cohort. Their study focused on the predictive validation of the criteria, so gaps remain regarding concurrent criterion validity and inter-rater reliability in this group of patients. Furthermore, the external applicability of Liu's findings to Latin American populations is limited due to sociodemographic and biological differences among patients. In Colombia, to our knowledge, no GLIM criterion validation studies have been conducted in any clinical setting^18^, reflecting the lack of local data to support its implementation.

Based on the scenario described, the objective of this research was to determine the concurrent and predictive criterion validity, and the inter-rater reliability of the GLIM criteria in Colombian patients undergoing cardiac surgery.

## Materials and Methods

**Study population and design**

This was a criterion validation study in a cohort of 51 adult patients (≥18 years) hospitalized with an indication for cardiac surgery, who agreed to participate voluntarily in the study, at a high-complexity health center in the city of Medellín-Colombia. Patients with the following were excluded: cancer, critical illness, neurocognitive disorders or altered state of consciousness that prevented the administration of the tests (e.g. major neurocognitive disorder, delirium), neuromuscular diseases (e.g. myasthenia gravis), and previous major surgery during the same hospitalization.

This research used census sampling: all participants who met the selection criteria during the study period (April–August 2024) were included. There is no unified criterion in the literature regarding the sample size required for evaluating the psychometric properties of health measurement scales. Different authors and scientific societies endorse the rule of thumb or the subject-item ratio, which suggests including at least 10 participants for each variable assessed on the scale^19^-^22^. In the case of the GLIM criteria, this corresponds to a minimum sample size of 50 participants.

**Ethical statement**

This study was conducted in accordance with the ethical principles for medical research involving human participants established by the Declaration of Helsinki (World Medical Association) and Colombian Resolution 008430 of 1993^23^. Based on the national resolution, it was classified as a minimal-risk study. The procedures, techniques, and instruments were approved by the Clinic's Ethics Committee (Minutes No. 216, January 25, 2024). Written informed consent was obtained from all participants.

**Nutritional diagnosis based on GLIM criteria**

The diagnosis of malnutrition by GLIM requires the combination of at least one phenotypic criterion and one etiological criterion. Phenotypic criteria include involuntary weight loss, low body mass index (BMI), and reduced muscle mass. Etiological criteria include: involuntary decrease in food intake (<50% of the recommended intake for more than one week or any reduction in intake for more than two weeks), presence of digestive disease or symptoms that negatively affect nutrient absorption, and disease-related inflammation (acute or chronic illness, infection, or injury that is usually associated with inflammatory activity). Subsequently, the degree of malnutrition (moderate or severe) is classified based on defined cut-off points for the phenotypic criteria Table 1^15^.

**Table 1:** Cut-off points for determining the severity of malnutrition

Degree of malnutrition	Unintentional weight loss	Body mass index	Reduction in muscle mass (calf circumference)*
Moderate malnutrition	>5-10% in the last 6 months or >10-20% in more than 6 months	<20 kg/m2 if <70 years <22 kg/m2 if ≥70 years	<34 cm male <33 cm female
Severe malnutrition	>10% in the last 6 months or >20% in more than 6 months	<18.5 kg/m2 if <70 years <20 kg/m2 if ≥70 years	<32 cm male <31cm female

Note: Adapted from Cederholm et al.^15^ and Gonzalez et al.^24^ *In this study, calf circumference was used as an indicator of muscle mass, considering its availability, portability, low cost, simplicity (minimal training) and quick application; using cut-off points validated against the Gold Standard of body composition ( dual energy X-ray absorptiometry)^24^.

**Evaluation of GLIM criteria**

**1. BMI:** The weight and height of the participants were measured using a digital scale with a built-in stadiometer (SECA 777), with sensitivities of 0.1 kg and 0.1 cm respectively. BMI was established using the formula: weight (kg) / height (m)2.

**2. Weight loss:** Usual weight was obtained from previous medical records provided the data was no older than 12 months. If this was unavailable, the patient was asked directly. The percentage of weight loss was calculated using the formula: (usual weight – current weight) / usual weight *100.

**3. Muscle mass:** Was estimated by measuring calf circumference (CC) with a Lufkin W606PM anthropometric tape (sensitivity 0.1 cm). The value obtained was classified according to the cut-off points suggested by González^24^ Table 1. In cases of excess weight, the CC value was adjusted by subtracting the estimated subcutaneous adipose tissue, as follows: -3 cm in overweight, -7 cm in grade I and II obesity and -12 cm in grade III-V obesity^24^. In addition, in cases of lower limb edema, 2 cm were subtracted for men and 1.6 cm for women^25^. For all anthropometric measurements, institutional protocols were followed.

Additionally, muscle strength was assessed using a CAMRY EH101 handheld digital dynamometer (sensitivity 0.1 kg), which has demonstrated excellent validity and reliability compared to the JAMAR dynamometer^26^, representing a cost-effective alternative. Following the diagnostic algorithm and cut-off points proposed at the second meeting of the European Working Group on Sarcopenia in Older Adults (EWGSOP2), patients were classified as having probable sarcopenia (dynapenia) when their strength was below 27 kg in men and 16 kg in women^27^. Patients with low strength and muscle mass were classified as having confirmed sarcopenia^27^. Physical performance was not assessed.

**4. Intake:** The patient was directly asked about their food intake over the past two weeks. If there had been an involuntary reduction, the percentage decrease was determined by using their usual diet as a reference (plate method).

**5. Digestive disease or symptoms:** The following diagnoses or background information recorded in the admission medical history were considered: bariatric surgery, short bowel syndrome, pancreatic insufficiency, gastroparesis, high output ostomy (>500 ml/24h), dysphagia and esophageal stenosis, or the presence of diarrheal disease, steatorrhea and vomiting in the last week^15^.

**6. Disease-related inflammation:** Was assumed to be part of decompensated cardiovascular disease and, when available, was confirmed with a C-reactive protein ≥3 mg/dL^28^.

**Nutritional diagnosis based on Subjective Global Assessment**

Subjective Global Assessment (SGA) is a universally accepted tool for the assessment of nutritional status, with application in various patient groups and care settings, and has therefore been traditionally used as a reference standard for the validation of new diagnostic methods^29^. The SGA is based on the evaluation of the patient's medical history (involuntary changes in weight and dietary intake, functional capacity, gastrointestinal symptoms with nutritional impact and metabolic stress associated with the disease) and on the nutrition-focused physical examination, which aims to identify subcutaneous fat loss, muscle wasting and the presence of edema or ascites^29^.

The creators of this tool suggest that, for diagnostic purposes, the assessment should focus on criteria for weight loss, decreased intake, and physical examination findings such as loss of subcutaneous fat and muscle wasting^29^,^30^. Based on the results, the patient is classified into three categories: A (well-nourished), B (moderately malnourished), and C (severely malnourished)^29^.

Although the classification of nutritional status is primarily qualitative, based on the predominance of signs and symptoms and the clinical judgment of the evaluator, the authors have suggested some descriptions for each category^30^:

**A. Well-nourished:** patients with weight loss <5% in the last six months or ≥5% but with recent weight gain (in the last two to four weeks) and improved appetite.

**B. Moderately malnourished:** patients with weight loss ≥5 - 10% in the last six months without recent gain or stabilization, decreased food intake and mild loss of subcutaneous tissue.

**C. Severely malnourished:** patients with weight loss >10% in the last six months, with severe loss of subcutaneous tissue and muscle wasting, often with edema.

For the application of the questionnaire, the guidelines and directives established by the authors were followed^30^.

**Inter-rater reliability**

Inter-rater reliability assesses the agreement between at least two trained evaluators, evaluating the same patients, using the same instrument, and on the same occasion^31^. For this purpose, all patients received a direct preoperative nutritional assessment, based on the GLIM criteria, by two clinical nutrition professionals, who performed the assessments independently and recorded the results in isolated databases to ensure blinding. The assessments were carried out on the same day.

**Concurrent criterion validation**

Concurrent criterion validity refers to the degree to which the results of a scale are valid when compared with the results of other certified instruments known as gold standards^31^. The GLIM validation guide^16^ established the SGA as a reference standard, so a third, blinded, and trained professional performed a new preoperative nutritional assessment according to the SGA, with a difference of 24 hours from the GLIM assessments.

**Predictive validation**

The predictive criterion validity determines the agreement between the scale result (malnutrition) with an event related to the phenomenon that may occur in the future (clinical outcome)^16^. According to the validation guide, to establish predictive validity in the hospital setting, outcomes such as hospital mortality, major complications, readmission and length of stay, which are expected to be associated with malnutrition, should be considered^16^. After surgery, patients were monitored through their medical records during their hospital stay and on day 30 post-discharge to determine the occurrence of outcomes. Follow-up was conducted in real time while the patient remained in the institution; therefore, missing data from the medical record were obtained by consulting directly with the attending physician, without requiring data entry. Furthermore, it was ensured that these were incidental outcomes and not prevalent ones.

The outcomes were segmented into one primary outcome and nine secondary outcomes. The secondary outcomes included the incidence of hospital mortality, sepsis and shock (from any cause) after surgery, surgical reintervention, length of hospital stay, length of stay in the intensive care unit (ICU), and operative times (surgical, clamping, and perfusion). Outcome confirmation was based on medical records.

For predictive validation, the primary outcome was hospital readmission within 30 days. This had to be readmission to the same health center and for causes associated with the surgery, according to the criteria of the emergency physician (e.g., surgical site infection, bleeding, wound dehiscence, pneumothorax, dyspnea, pleural effusion, hypotension, and uncontrolled pain).

**Covariates**

The following variables were obtained from clinical records and pre-anesthetic assessment: age, sex, smoking habit, alcohol consumption, left ventricular ejection fraction (%LVEF), functional classification New York Health Association (NYHA), cardiac surgery mortality score Society of Thoracic Surgeons (STS), nutritional support, number of chronic diseases, hemoglobin, white blood cell count, neutrophil-to-lymphocyte ratio, and creatinine. For laboratory results, the time window was limited to 7 days prior to the nutritional assessment. When multiple results were available, the most recent was included. Patients were also classified according to the type of surgery and the valves operated on.

The information was recorded in a database designed in Microsoft Excel, which included validations and restrictions to minimize data entry errors. The tool was evaluated through a pilot test with 10 participants from the study population. In case of doubts or inconsistencies, the research team verified the information directly with the treating professional. This was possible thanks to the prospective design of the research.

**Statistical analysis**

Analyses were performed using the Jamovi statistical platform, version 2.3.21 (The Jamovi Project 2023), and the R statistical software, version 4.4.0 (R Core Team 2024). Quantitative variables are summarized using mean and standard deviation or median and interquartile range. Normality was assessed using the Shapiro-Wilk test. Categorical variables are summarized using absolute and relative frequencies. To compare baseline characteristics according to nutritional status, the student’s t-test or Mann-Whitney U test was used for quantitative variables, and the chi-square test of independence or Fisher's exact test was used for qualitative variables.

Inter-rater agreement was determined using Cohen's kappa index weighted by squared weights for ordinal scales. Classification was based on the Landis and Koch criteria^32^. The cutoff point for determining inter-rater reliability was >0.8^16^. For concurrent criterion validity, the simple kappa index was used, and diagnostic performance measures (sensitivity, specificity, predictive values, and diagnostic odds ratio). Validation was achieved if both sensitivity and specificity were greater than 80%^16^.

For predictive criterion validation, a robust variance generalized linear regression model (Poisson family) was performed to obtain simple and adjusted relative risks (RR). Predictive validity was concluded if the effect size was ≥ 2.0^16^. The assumptions of this model were tested, and the goodness of fit was assessed using metrics such as deviance and pseudo-R². A multiple linear regression model was also performed to establish the association between length of hospital stay (dependent) and muscle mass (independent). The model assumptions were corroborated, and the goodness of fit was assessed using R² and the F-test for overall significance. The adjustment variables for both models were selected based on clinical criteria. All collected data are freely available for consultation on Mendeley Data^33^.

## Results

**Characterization of the study population**

The baseline characteristics of the participants are described in Table 2. According to nutritional status, there was a statistical difference in the proportion of confirmed sarcopenia, being significantly (p=0.001) higher in people with malnutrition (46.43%) compared to those with adequate nutritional status (4.35%).

**Table 2:** Sociodemographic and Clinical Characterization According to Nutritional Status

Basic features	General (n=51) % (n)	Well nourished (n=23) % (n)	Malnutrition (n=28) % (n)	p-value*
Sex, male	62.75 (32)	65.22 (15)	60.71 (17)	0.74 ‡
Age (years)^a^	64.14 ± 11.00	62.87 ± 11.75	65.18 ± 10.45	0.46 †
LVEF^b^	55.00 [50.00;60.00]	55.00 [50.00; 60.00]	55.00 [50.00; 60.00]	0.66
STS^b^	1.32 [0.80; 2.49]	1.30 [0.76; 1.85]	1.45 [0.93; 2.71]	0.34
Nutritional support	19.61 (10)	13.04 (3)	25.00 (7)	0.48
Chronic diseases^b^	3.00 [2.00; 4.00]	3.00 [2.00; 4.00]	3.00 [2.00; 5.00]	0.93
Hemoglobin (g/ dL)^a^	14.36 ± 1.72	14.70 ± 1.68	14.09 ± 1.73	0.22 †
Leukocytes (103 uL)^a^	8.67 ± 2.54	8.19 ± 2.26	9.07 ± 2.73	0.24 †
Neutrophil/ lymphocyte ratio^b^	2.56 [1.78; 3.56]	2.73 [1.90; 3.36]	2.55 [1.77; 3.58]	0.90
Creatinine (g/ dL)^b^	0.90 [0.77; 1.18]	0.92 [0.78; 1.10]	0.90 [0.75; 1.21]	0.93
Smoker asset	13.73 (7)	8.70 (2)	17.86 (5)	0.46
Alcohol consumption	11.76 (6)	17.39 (4)	7.14 (2)	0.39
Grip strength (kg)^b^	27.45 [20.05;33.35]	27.15 [21.20; 41.15]	23.00 [19.20; 27.90]	0.06
Probable sarcopenia	43.14 (22)	34.78 (8)	50.00 (14)	0.39 ‡
Confirmed sarcopenia	27.45 (14)	4.35 (1)	46.43 (13)	0.001
Type of surgery				0.14
Revascularization myocardium	65.96 (31)	54.55 (12)	76.00 (19)	
Valvular	19.15 (9)	31.82 (7)	8.00 (2)	
Combined	14.89 (7)	13.64 (3)	16.00 (4)

a:Mean ± standard deviation; b:Median [interquartile range]; *Mann-Whitney U test for quantitative variables and Fisher's exact test for categorical variables, unless otherwise indicated. † Student's t-test for independent samples; ‡ Chi-square test of independence (Sex, probable sarcopenia) or Fisher's exact test (for all others). LVEF: Left ventricular ejection fraction; STS: Society of Thoracic Surgeons operative mortality score.

**Preoperative prevalence of malnutrition according to GLIM criteria**

More than half of the participants presented with some degree of malnutrition (54.90%). Reduced muscle mass was the most frequent phenotypic criterion; 45.10% of patients exhibited moderate or severe muscle depletion. The predominant etiological criterion was reduced food intake (37.25%). Inflammation was a constant finding.

**Inter-rater reliability**

An almost perfect agreement was found in the nutritional diagnosis by GLIM between the two professionals (Kappa 0.94, 95% CI 0.88-0.99, p <0.001) verifying the inter-rater reliability of these criteria.

**Concurrent validity**

Nutritional diagnosis using GLIM showed substantial agreement with diagnosis using SGA (Kappa 0.64, 95% CI 0.44–0.85, p <0.001). GLIM identified 88.00% of patients with malnutrition (Sensitivity 88.00, 95% CI 68.78–97.45) and 76.92% of well-nourished patients (Specificity 76.92, 95% CI 56.35–91.03). Diagnostic accuracy was 82.35%, and the positive and negative predictive values were 78.57% and 86.96%, respectively. The probability of correctly diagnosing nutritional status with GLIM is 24.44 times the probability of making a mistake (ORd 24.44, 95% CI 5.39–110.92).

**Association between nutritional status and clinical outcomes**

**Figure 1:**
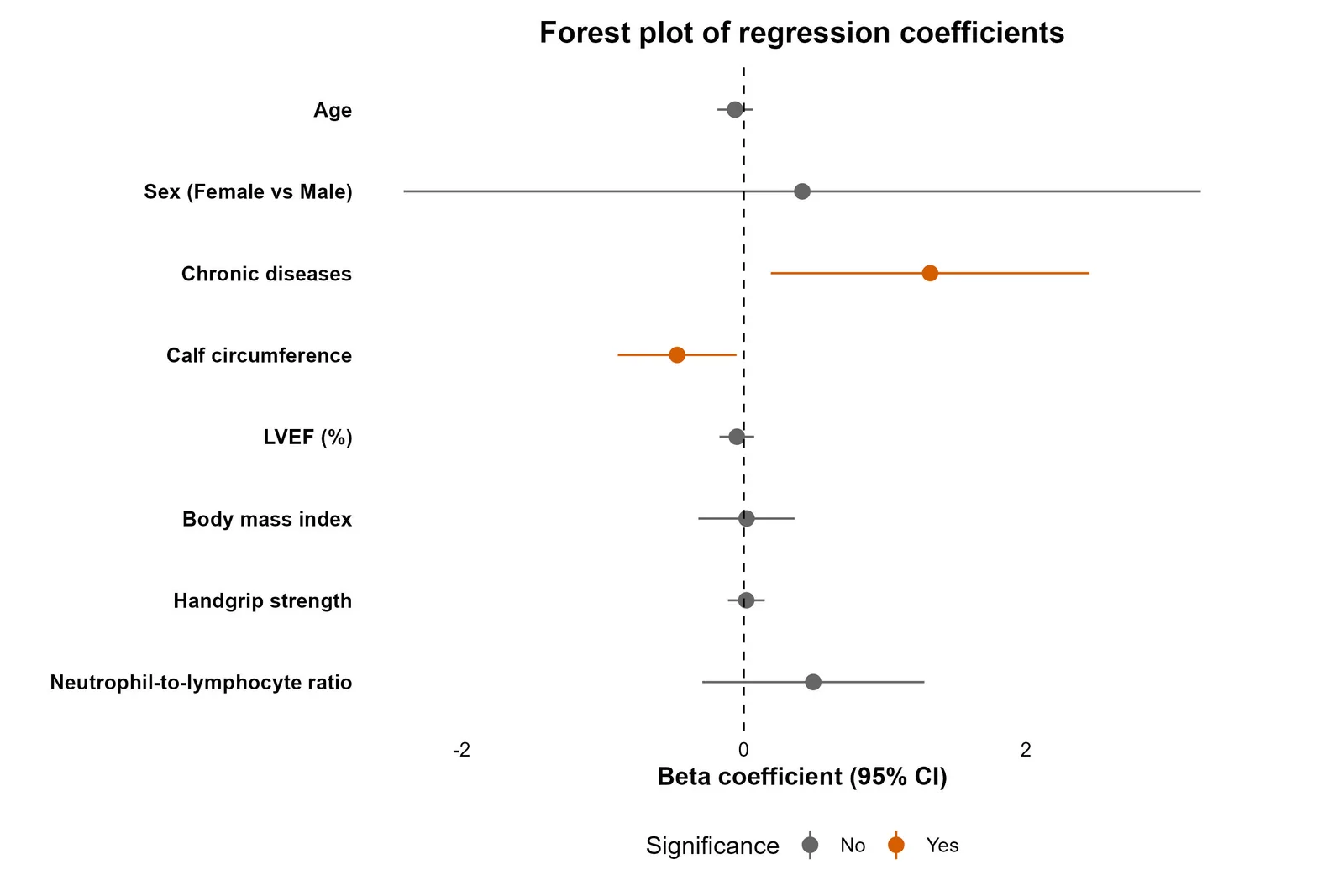
Association between calf circumference and days of hospital stay

Of the 51 patients evaluated, 4 declined surgical intervention (n=47): 3 with malnutrition and 1 with adequate nutritional status. No statistically significant differences were observed in the incidence of secondary outcomes according to overall nutritional status Table 3. However, an inverse linear association was found between calf circumference and length of hospital stay, which remained after adjusting the model (β -0.47, 95% CI -0.89 to -0.05, p 0.03) Figure 1. It was also reported that for each concomitant chronic disease, the length of hospital stays increased by 1.3 days (β 1.32, 95% CI 0.19 to 2.45, p 0.02) Figure 1.

**Table 3:** Postoperative outcomes according to nutritional status

Clinical outcomes	General (n=47) % (n)	Well nourished (n=22) % (n)	Malnutrition (n=25) % (n)	p-value*
Days of stay in ICU^b^	3.00 [2.00; 4.00]	3.0 [1.25; 4.00]	3.00 [2.00; 5.00]	0.61
Days of hospital stay^b^	6.00 [5.00; 8.50]	6.00 [5.00; 7.00]	7.00 [5.00; 10.00]	0.11
Hospital mortality	6.38 (3)	9.09 (2)	4.00 (1)	0.59
Shock for any reason	21.28 (10)	31.82 (7)	12.00 (3)	0.15
Surgical reintervention	6.38 (3)	9.09 (2)	4.00 (1)	0.33
Sepsis	8.51 (4)	0.00 (0)	16.00 (4)	0.11
Surgical time (min)^b^	300.00 [300.00;330.00]	300.00 [247.50; 330.00]	300.00 [300.00; 300.00]	0.33
Infusion time (min)^b^	124.00 [93.00; 139.00]	130.50 [72.00; 144.25]	123.00[101.00; 139]	1.00
Clamp time (min)^b^	103.00 [70.00; 123.00]	109.00 [58.75; 128.25]	97.00 [84.00; 120.00]	0.84

b Median [interquartile range], min: minutes. *Mann-Whitney U test for quantitative variables and Fisher's exact test for qualitative variables.

Each circle in the graph represents the regression coefficient (β), which indicates the estimated change in length of hospital stay for each unit change in each independent variable. Positive coefficients reflect an increase in length of stay, while negative coefficients indicate a decrease. The horizontal lines show the 95% confidence intervals. The vertical line plotted at zero represents the point of non-significance; if an interval includes zero, it suggests no statistical association between the variables. Fitted model: normality of residuals (Shapiro-Wilk p = 0.52); independence of errors (DW statistic 2.25, p = 0.42); homoscedasticity (Breusch - Pagan p = 0.06); all variance inflation factors were less than two. -R2 0.440. RMSE 2.95. Global Model Test p = 0.01. Adjusted for age, sex, number of chronic diseases, left ventricular ejection fraction (%), body mass index, grip strength, and neutrophil-lymphocyte index.

**Predictive criterion validity**

GLIM malnutrition acted as a risk factor for hospital readmission at 30 days. Patients with malnutrition had a 1.59 times greater risk of readmission due to post-surgical complications than patients with adequate nutritional status, confirming the predictive criterion validity (RRa 2.59, 95% CI 1.09 – 7.02, p 0.04) Table 4.

**Table 4:** Association between nutritional status and hospital readmission at 30 days

Variable	RR crude 95% CI	RR adjusted 95% CI	p-value
Nutritional status			
Well nourished	1	1	
Malnutrition	2.45 (1.15 - 5.77)	2.59 (1.09 - 7.02)	0.04
Age	0.97 (0.95 - 1.01)	0.97 (0.93 - 1.01)	0.16
Sex			
Female	1	1	
Male	1.19 (0.57 - 2.41)	1.19 (0.42 - 3.19)	0.73
% LVEF	0.99 (0.95 - 1.04)	0.99 (0.95 - 1.04)	0.71
Chronic diseases	1.05 (0.82 - 1.34)	1.16 (0.86 - 1.54)	0.32
Grip strength	0.99 (0.96 - 1.03)	1.01 (0.95 - 1.06)	0.76

RR: Relative Risks - Adjusted for age, sex, number of chronic diseases, preoperative left ventricular ejection fraction (%) and grip strength. All variance inflation factors were less than 2. Pseudo-R2 0.169 Deviance 27. LVEF: Left ventricular ejection fraction.

## Discussion

This study demonstrated the inter-rater reliability and predictive validity of the GLIM operational criteria in a group of Colombian patients undergoing cardiac surgery. Although the specificity threshold for concurrent criterion validity was not reached, the overall results suggest acceptable diagnostic performance for malnutrition.

Few studies have explored the validity of GLIM in the context of cardiac surgery. A retrospective analysis conducted by Thomas et al.^34^ in 224 patients from a vascular surgery unit in Australia, found moderate diagnostic agreement (Kappa 0.42) between GLIM and patient-generated subjective global assessment (PG-SGA), with a sensitivity of 73.7% (95% CI 52.8-94.8) and a specificity of 80.6% (95% CI 75.2-86.0%). In 119 Spanish adults hospitalized for acute medical conditions, including a subgroup of patients with cardiovascular disease (9.2%), substantial agreement (Kappa 0.64, 95% CI 0.50–0.79) was observed between GLIM and SGA, with a reported sensitivity of 78% (95% CI 64.0–88.5) and specificity of 86.2% (95% CI 75.3–93.5)^35^. The studies described present findings moderately like those of the present investigation. None managed to demonstrate concurrent criterion validity by not reaching the sensitivity and specificity thresholds (>80%) (15) . However, a prospective cohort study conducted in five high-complexity hospitals in Brazil (n=601), demonstrated the concurrent validity of the GLIM criteria versus the SGA, in hospitalized adults with chronic conditions (12.9% for cardiovascular disease) and undergoing surgical interventions, with a sensitivity of 86.6% and a specificity of 81.6%^36^.

The proportion of malnutrition determined by GLIM differs from that obtained by SGA or PG-SGA^34^–^36^. This is primarily due to the methods used to measure muscle mass, a predominant criterion in the diagnosis of malnutrition^37^ that is closely related to health outcomes^38^. SGA is based on the detection of qualitative signs of muscle depletion through physical examination, which can underestimate low muscle mass, especially in patients with abundant subcutaneous adipose tissue^39^. In contrast, GLIM suggests the use of body composition technologies such as bioelectrical impedance, computed tomography, ultrasound, and dual-energy X-ray absorptiometry (DXA), or, failing that, anthropometric measurements such as calf circumference, which has been validated against these methods and for which cut-off points and adjustments have been determined to improve its validity^24^,^39^,^40^.

Preoperative muscle mass is a predictor of hospital stay in cardiac surgery patients. Zuckerman et al.^41^ reported in a cohort of older adults, that for every centimeter increase in psoas muscle area, postoperative hospital stay decreased by 2.35 days (β –2.35, 95% CI –4.48 to –0.22). In patients undergoing transcatheter aortic valve replacement (TAVR), an increase in skeletal muscle index was found to be associated with a one-day reduction in hospital stay (p 0.03)^42^, and Shibasaki et al.^43^ demonstrated in cardiac surgery patients that sarcopenia (decreased muscle mass and function) was the factor most strongly associated with prolonged stay (>20 days) (OR 2.507, 95% CI 1.138 – 5.521, p <0.05). Our study also revealed an inverse association between muscle mass, measured by calf circumference, and postoperative stay. These findings are consistent with those reported by Tarnowski^44^ in hospitalized patients, who highlighted an increased probability of prolonged hospitalization (>16 days) in patients with decreased calf circumference (OR 1.59, 95% CI 1.07 – 2.36, p 0.023).

Patients undergoing cardiac surgery are prone to muscle wasting during hospitalization due to the inflammatory response to surgical stress and factors such as immobility or deterioration of functional status, the need for mechanical ventilation, and malnutrition, exacerbating deficits present at admission^14^,^45^. In the first postoperative week, a 16% reduction in the transverse area of the rectus femoris and a 24% reduction in the pennation angle have been described^45^. Muscle waste in the first week after cardiac surgery prolongs the ICU stay and the need for mechanical ventilation^46^.

Muscle mass is also part of the operational definition of sarcopenia^27^,^47^, understood as a progressive and generalized musculoskeletal disorder, associated with adverse health outcomes^27^. Malnutrition has been recognized as one of the main predictors of sarcopenia (HR 3.23, 95% CI 1.73 – 6.05) and severe sarcopenia (HR 2.87, 95% CI 1.25 – 6.56)^48^ and both conditions can coexist in patients with cardiovascular disease, increasing the risk of death in the medium and long term^49^,^50^. Therefore, GLIM considers the association between nutritional status and syndromes such as sarcopenia and frailty, theoretically related to malnutrition, as an indirect form of validation, which it defines as convergent construct validity, determined through hypothesis testing (X2 p <0.05 if n: <200 or p<0.01 if n: ≥200)^16^. This study provides theoretical evidence that supports the construct validity of GLIM in the context of cardiac surgery, by reaffirming the association between malnutrition and sarcopenia (p <0.001, difference of proportions 0.421, 95% CI 0.21 – 0.62).

Regarding the predictive criterion validity, hospital readmission within 30 days was selected as the primary outcome^16^, considering the low incidence of postoperative outcomes during the hospital stay, which is related to short hospital observation times after the intervention (median 6 days). Cardiac surgery patients tend to have short stays in both the ICU and the hospital, which can make it difficult to identify intermediate and late complications^46^,^51^.

To our knowledge, only one study has explored the predictive validity of GLIM in cardiac surgery. Liu et al.^11^ conducted an observational study in 603 adult patients from the cardiothoracic surgery department of Tenth People's Hospital in Shanghai, undergoing myocardial revascularization or valve surgery, showing that malnutrition was associated with postoperative complications (Clavien -Dindo ≥2) (OR 1.66, 95% CI 1.063–2.594, p 0.026) and lower overall survival at three-year follow-up (HR 2.339, 95% CI 1.504–3.637, p <0.001). These findings were confirmed in a post hoc analysis in older adults (≥65 years) (n=401), although in this group the threshold (HR, RR, OR >2) for the predictive criterion was not reached (HR 1.862, 95% CI 1.171–2.962, p 0.009)^52^.

Liu's study did not describe hospital readmission; however, in internal medicine patients, GLIM malnutrition has been shown to increase the 30-day readmission risk by 66% (RR 1.66, 95% CI 1.06–2.62, p 0.026)^53^. Furthermore, in a cohort of cancer patients (n = 2801), in which the validity of three diagnostic malnutrition scales was compared, GLIM demonstrated the best predictive capacity for unplanned admission and 30-day readmission (OR 1.78, 95% CI 1.34–2.35, p < 0.001)^54^. The literature highlights an increased risk of readmission in malnourished individuals, which we have reported in our study.

No studies have been identified that evaluate the inter-rater reliability of GLIM in cardiac surgery. However, in patients with gastrointestinal cancer (n=1115), Tan et al.^55^ reported substantial agreement between independent evaluators when all diagnostic categories were included (Kappa 0.78, 95% CI 0.74–0.82). Similarly, in patients with head and neck cancer (n=188), almost perfect agreement (Kappa 0.985) was observed between trained dietitians^56^. Together with our results, these findings support the reproducibility and clinical applicability of GLIM.

Given this scenario, the need for timely detection of malnutrition in cardiac surgical patients through methodologies such as GLIM, and the implementation of nutritional prehabilitation programs is highlighted, with the objectives of mitigating the nutritional impact of the disease and the surgical procedure, and optimizing health outcomes, as suggested by the ERAS guidelines^57^. An example of this is the preoperative administration of oral nutritional supplements high in protein or enriched with immunonutrients, which appear to reduce weight loss, the rate of infectious and non-infectious complications and hospital stay^58^,^59^.

**Strengths and limitations**

This study has some limitations. First, the sample size was small, which may have affected the accuracy of the results and the statistical power needed to identify other associations. Second, the diagnosis of outcomes was based on the treating physician's judgment. Adherence to standardized diagnostic criteria or clinical practice guidelines was not assessed. Third, this was a single-center study of patients with severe coronary or valvular heart disease, most of whom were admitted in the context of an acute coronary syndrome. This limits the generalizability of the results and reduces the representation of patients with milder forms of the disease or those scheduled for elective surgery, in whom the prevalence of malnutrition may be lower. Fourth, hospital readmission was only recorded for the institution where the surgery was performed. Some patients may have experienced postoperative complications and sought care at other healthcare facilities (loss to follow-up). Finally, the nutritional assessment included criteria such as weight loss and reduced food intake, both of which are subject to potential bias. Usual weight may be inaccurate due to imprecise patient recall or unreliable previous records, and the reduction in intake was based on self-report, which introduces subjectivity.

Among the strengths of this study, it is noteworthy that, to the authors' knowledge, it is the first to jointly and prospectively evaluate the concurrent and predictive criterion validity, as well as the inter-rater reliability, of the GLIM criteria in cardiac surgery patients. Furthermore, it is the first GLIM criterion validation study in Colombian patients. Nutritional status assessment was performed by trained professionals (primary source) using validated techniques and cut-off points. The prospective nature of this research ensured the temporality of the events (malnutrition – outcome).

**Recommendations for future research**

Long-term longitudinal cohort studies with larger, multicenter samples are suggested to improve the generalizability of the findings. Furthermore, incorporating body composition technologies such as DXA is recommended to achieve a more objective nutritional and functional assessment, which would also allow for the exploration of other phenotypes such as sarcopenic obesity. Finally, local, controlled, randomized clinical trials are needed to evaluate whether early nutritional interventions optimize clinical and economic outcomes in this patient group and to determine the type and duration of such interventions.

## Conclusions

The GLIM criteria are reliable for diagnosing malnutrition and have predictive validity for relevant clinical outcomes in patients undergoing cardiac surgery, supporting their utility and applicability in clinical practice. Although the established specificity threshold for concurrent validation was not reached, the overall diagnostic capacity of these criteria was satisfactory. Consequently, GLIM can be considered a tool for nutritional diagnosis in this population, as well as for the timely identification of patients eligible for nutritional prehabilitation programs, with the aim of optimizing their clinical outcomes.

## References

[1] Cederholm  T, Bosaeus  I (2024). Malnutrition in Adults. N Engl J Med.

[2] Schuetz  P, Seres  D, Lobo  DN, Gomes  F, Kaegi-Braun  N, Stanga  Z (2021). Management of disease-related malnutrition for patients being treated in hospital. Lancet.

[3] Jobim Milanez  DS, Razzera  EL, da Silveira Knobloch  I, Lima  J, Bernardes  S, Silva  FM (2023). A scoping review on the GLIM criteria for malnutrition diagnosis: Understanding how and for which purpose it has been applied in studies on hospital settings. Clin Nutr.

[4] Correia  MITD, Perman  MI, Waitzberg  DL (2017). Hospital malnutrition in Latin America: A systematic review. Clin Nutr.

[5] Uenishi  M, Song  P (2025). New diagnostic code “5B72 Undernutrition in Adults” approved for inclusion in the 11th Revision of the International Classification of Diseases (ICD-11). Drug Discov Ther.

[6] Thanapholsart  J, Khan  E, Ismail  TF, Lee  GA (2023). The complex pathophysiology of cardiac cachexia: A review of current pathophysiology and implications for clinical practice. Am J Med Sci.

[7] Savino Lloreda  P, Posada Álvarez  C, López Daza  D (2020). Nutrición aplicada en patologías crónicas.

[8] Mubashir  T, Balogh  J, Breland  E, Rumpel  D, Waheed  MA, Lai  H (2022). Risk Factors and Outcomes of Protein-Calorie Malnutrition in Chronic Heart Failure Patients Undergoing Elective Cardiac Surgery. Cureus.

[9] Unosawa  S, Taoka  M, Osaka  S, Yuji  D, Kitazumi  Y, Suzuki  K (2019). Is malnutrition associated with postoperative complications after cardiac surgery?. J Card Surg.

[10] Chermesh  I, Hajos  J, Mashiach  T, Bozhko  M, Shani  L, Nir  RR (2014). Malnutrition in cardiac surgery: food for thought. European Journal Preventive Cardiology.

[11] Liu  Z, Shen  Z, Zang  W, Zhou  J, Yu  Z, Zhang  P (2022). Development and Validation of Global Leadership Initiative on Malnutrition for Prognostic Prediction in Patients Who Underwent Cardiac Surgery. Nutrients.

[12] Drover  JW, Cahill  NE, Kutsogiannis  J, Pagliarello  G, Wischmeyer  P, Wang  M (2010). Nutrition Therapy for the Critically Ill Surgical Patient: We Need To Do Better!. J Parenter Enter Nutr.

[13] Rahman  A, Agarwala  R, Martin  C, Nagpal  D, Teitelbaum  M, Heyland  DK (2016). Nutrition Therapy in Critically Ill Patients Following Cardiac Surgery: Defining and Improving Practice. J Parenter Enter Nutr.

[14] Stoppe  C, Goetzenich  A, Whitman  G, Ohkuma  R, Brown  T, Hatzakorzian  R (2017). Role of nutrition support in adult cardiac surgery: a consensus statement from an International Multidisciplinary Expert Group on Nutrition in Cardiac Surgery. Crit Care.

[15] Cederholm  T, Jensen  GL, Correia  MITD, Gonzalez  MC, Fukushima  R, Higashiguchi  T (2019). GLIM criteria for the diagnosis of malnutrition – A consensus report from the global clinical nutrition community. J Cachexia Sarcopenia Muscle.

[16] Keller  H, Van Der Schueren  MAE, GLIM  Consortium, Jensen  GL, Barazzoni  R, Compher  C (2020). Global Leadership Initiative on Malnutrition (GLIM): Guidance on Validation of the Operational Criteria for the Diagnosis of Protein-Energy Malnutrition in Adults. J Parenter Enter Nutr.

[17] Correia  MITD, Tappenden  KA, Malone  A, Prado  CM, Evans  DC, Sauer  AC (2022). Utilization and validation of the Global Leadership Initiative on Malnutrition (GLIM): A scoping review. Clin Nutr.

[18] Pérez  A, Díaz Muñoz  GA, Maza Moscoso  CP, Castro Muñoz  MG, Canicoba  ME, Gonzalez  MC (2022). Modelo de proceso de cuidado nutricional: consenso para Latinoamérica. Rev Nutr Clínica Metab.

[19] Roco Videla  Á, Hernández Orellana  M, Silva González  O (2021). ¿Cual es el tamaño muestral adecuado para Validar un cuestionario?. Nutr Hosp.

[20] Morgado  FFR, Meireles  JFF, Neves  CM, Amaral  ACS, Ferreira  MEC (2017). Scale development: ten main limitations and recommendations to improve future research practices. Psicol Reflex Crit.

[21] Anthoine  E, Moret  L, Regnault  A, Sébille  V, Hardouin  JB (2014). Sample size used to validate a scale: a review of publications on newly-developed patient reported outcomes measures. Health Qual Life Outcomes.

[22] International Test Commission (2017). The ITC Guidelines for Translating and Adapting Tests (Second edition).

[23] Ministerio de Salud de Colombia (1993). Resolución 8430 de 1993, por la cual se establecen las normas científicas, técnicas y administrativas para la investigación en salud.

[24] Gonzalez  MC, Mehrnezhad  A, Razaviarab  N, Barbosa-Silva  TG, Heymsfield  SB (2021). Calf circumference: cutoff values from the NHANES 1999–2006. Am J Clin Nutr.

[25] Ishida  Y, Maeda  K, Nonogaki  T, Shimizu  A, Yamanaka  Y, Matsuyama  R (2019). Impact of edema on length of calf circumference in older adults. Geriatr Gerontol Int.

[26] Huang  L, Liu  Y, Lin  T, Hou  L, Song  Q, Ge  N (2022). Reliability and validity of two hand dynamometers when used by community-dwelling adults aged over 50 years. BMC Geriatr.

[27] Cruz-Jentoft  AJ, Bahat  G, Bauer  J, Boirie  Y, Bruyère  O, Cederholm  T (2019). Sarcopenia: revised European consensus on definition and diagnosis. Age Ageing.

[28] Jensen  GL, Cederholm  T, Ballesteros-Pomar  MD, Blaauw  R, Correia  MITD, Cuerda  C (2024). Guidance for assessment of the inflammation etiologic criterion for the GLIM diagnosis of malnutrition: A modified Delphi approach. J Parenter Enter Nutr.

[29] Barbosa-Silva  MCG, Barros  AJ (2006). Indications and limitations of the use of subjective global assessment in clinical practice: an update. Curr Opin Clin Nutr Metab Care.

[30] Detsky  AS, Smalley  PS, Chang  J (1994). Is this patient malnourished. JAMA.

[31] Luján-Tangarife  JA, Cardona-Arias  JA (2015). Construcción y validación de escalas de medición en salud: revisión de propiedades psicométricas. Archivos de Medicina.

[32] Landis  JR, Koch  GG (1977). The measurement of observer agreement for categorical data. Biometrics.

[33] Londoño-Pereira  M (2025). Validación criterios GLIM Cirugía Cardíaca. Mendeley Data, V1.

[34] Thomas  J, Delaney  C, Miller  M (2023). The ability of the Global Leadership Initiative on Malnutrition (GLIM) to diagnose protein–energy malnutrition in patients requiring vascular surgery: a validation study. Br J Nutr.

[35] Fontane  L, Reig  MH, Garcia-Ribera  S, Herranz  M, Miracle  M, Chillaron  JJ (2023). Validity and Applicability of the Global Leadership Initiative on Malnutrition (GLIM) Criteria in Patients Hospitalized for Acute Medical Conditions. Nutrients.

[36] Brito  JE, Burgel  CF, Lima  J, Chites  VS, Saragiotto  CB, Rabito  EI (2021). GLIM criteria for malnutrition diagnosis of hospitalized patients presents satisfactory criterion validity: A prospective cohort study. Clin Nutr.

[37] Boslooper-Meulenbelt  K, Van Vliet  IMY, Gomes-Neto  AW, De Jong  MFC, Bakker  SJL, Jager-Wittenaar  H (2021). Malnutrition according to GLIM criteria in stable renal transplant recipients: Reduced muscle mass as predominant phenotypic criterion. Clin Nutr.

[38] Orsso  CE, Montes-Ibarra  M, Findlay  M, Van Der Meij  BS, De Van Der Schueren  MAE, Landi  F (2022). Mapping ongoing nutrition intervention trials in muscle, sarcopenia, and cachexia: a scoping review of future research. J Cachexia Sarcopenia Muscle.

[39] Compher  C, Cederholm  T, Correia  MITD, Gonzalez  MC, Higashiguch  T, Shi  HP (2022). Guidance for assessment of the muscle mass phenotypic criterion for the Global Leadership Initiative on Malnutrition diagnosis of malnutrition. J Parenter Enter Nutr.

[40] Prado  CM, Landi  F, Chew  STH, Atherton  PJ, Molinger  J, Ruck  T (2022). Advances in muscle health and nutrition: A toolkit for healthcare professionals. Clin Nutr.

[41] Zuckerman  J, Ades  M, Mullie  L, Trnkus  A, Morin  JF, Langlois  Y (2017). Psoas Muscle Area and Length of Stay in Older Adults Undergoing Cardiac Operations. Ann Thorac Surg.

[42] Dahya  V, Xiao  J, Prado  CM, Burroughs  P, McGee  D, Silva  AC (2016). Computed tomography–derived skeletal muscle index: A novel predictor of frailty and hospital length of stay after transcatheter aortic valve replacement. Am Heart J.

[43] Shibasaki  I, Ouchi  M, Fukuda  T, Tsuchiya  G, Ogawa  H, Takei  Y (2022). Effect of sarcopenia on hospital stay from post cardiac surgery to discharge. IJC Heart Vasc.

[44] Tarnowski  M, Stein  E, Marcadenti  A, Fink  J, Rabito  E, Silva  FM (2020). Calf Circumference Is a Good Predictor of Longer Hospital Stay and Nutritional Risk in Emergency Patients: A Prospective Cohort Study. J Am Coll Nutr.

[45] Buitrago  NDC, Gallego  DT, Pérez  MCF, Cardona  CAQ, Botero  CC (2024). Assessment of quadriceps muscle mass by ultrasound in the postoperative period of cardiac surgery. The Ultrasound Journal.

[46] Dimopoulos  S, Raidou  V, Elaiopoulos  D, Chatzivasiloglou  F, Markantonaki  D, Lyberopoulou  E (2020). Sonographic muscle mass assessment in patients after cardiac surgery. World Journal Cardiology.

[47] Kirk  B, Cawthon  PM, Arai  H, Ávila-Funes  JA, Barazzoni  R, Bhasin  S (2024). The Conceptual Definition of Sarcopenia: Delphi Consensus from the Global Leadership Initiative in Sarcopenia (GLIS). Age and Ageing.

[48] Beaudart  C, Sanchez-Rodriguez  D, Locquet  M, Reginster  JY, Lengelé  L, Bruyère  O (2019). Malnutrition as a Strong Predictor of the Onset of Sarcopenia. Nutrients.

[49] Ikeda  S, Kodama  A, Kawai  Y, Tsuruoka  T, Sugimoto  M, Niimi  K (2022). Preoperative sarcopenia and malnutrition are correlated with poor long-term survival after endovascular abdominal aortic aneurysm repair. Surgery Today.

[50] Ohori  K, Yano  T, Katano  S, Nagaoka  R, Numazawa  R, Yamano  K (2024). Coexistence of sarcopenia and self-reported weight loss is a powerful predictor of mortality in older patients with heart failure. Geriatr Gerontol Int.

[51] Lee  JJR, Srinivasan  R, Ong  CS, Alejo  D, Schena  S, Shpitser  I (2023). Causal determinants of postoperative length of stay in cardiac surgery using causal graphical learning. J Thorac Cardiovasc Surg.

[52] Liu  Z, Zang  W, Zhang  P, Shen  Z (2023). Prognostic implications of Global Leadership Initiative on Malnutrition–defined malnutrition in older patients who underwent cardiac surgery in China. Surgery.

[53] Cruz  PL, Soares  BLDM, Da Silva  JE, Lima E Silva  RRD (2022). Clinical and nutritional predictors of hospital readmission within 30 days. Eur J Clin Nutr.

[54] Poulter  S, Steer  B, Baguley  B, Edbrooke  L, Kiss  N (2021). Comparison of the GLIM, ESPEN and ICD-10 Criteria to Diagnose Malnutrition and Predict 30-Day Outcomes: An Observational Study in an Oncology Population. Nutrients.

[55] Tan  S, Wang  J, Zhou  F, Tang  M, Xu  J, Zhang  Y (2022). Validation of GLIM malnutrition criteria in cancer patients undergoing major abdominal surgery: A large-scale prospective study. Clin Nutr.

[56] Steer  B, Loeliger  J, Edbrooke  L, Deftereos  I, Laing  E, Kiss  N (2020). Malnutrition Prevalence according to the GLIM Criteria in Head and Neck Cancer Patients Undergoing Cancer Treatment. Nutrients.

[57] Engelman  DT, Ben Ali  W, Williams  JB, Perrault  LP, Reddy  VS, Arora  RC (2019). Guidelines for Perioperative Care in Cardiac Surgery: Enhanced Recovery After Surgery Society Recommendations. JAMA Surg.

[58] Hill  A, Arora  RC, Engelman  DT, Stoppe  C (2020). Preoperative Treatment of Malnutrition and Sarcopenia in Cardiac Surgery: New Frontiers. Crit Care Clin.

[59] Lopez-Delgado  JC, Muñoz-del Rio  G, Flordelís-Lasierra  JL, Putzu  A (2019). Nutrition in Adult Cardiac Surgery: Preoperative Evaluation, Management in the Postoperative Period, and Clinical Implications for Outcomes. J Cardiothorac Vasc Anesth.

